# Evaluation of Adverse Effects and Tolerability of Dietary Ginger Supplementation in Patients With Functional Dyspepsia

**DOI:** 10.1016/j.curtheres.2025.100792

**Published:** 2025-04-14

**Authors:** Lemlem Gebremariam Aregawi, Csiki Zoltan

**Affiliations:** a Institute of Nutrition, Faculty of Medical and Health Sciences, University of Debrecen, Debrecen, Hungary; b Department of Internal Medicine, Faculty of Medicine, University of Debrecen, Debrecen, Hungary; c Department of Public Health, College of Medicine and Health Sciences, Adigrat University, Adigrat, Ethiopia

**Keywords:** Adverse effects, Clinical trial, Functional dyspepsia, Ginger supplementation, Tolerability

## Abstract

**Background:**

Functional dyspepsia (FD) is a prevalent upper gastrointestinal disorder characterized by chronic or recurrent symptoms, including epigastric pain, bloating, and nausea. Ginger (*Zingiber officinale*), a natural dietary supplement traditionally used to relieve gastrointestinal discomfort, has limited evidence regarding its safety and tolerability in patients with FD.

**Objective:**

To evaluate the safety, tolerability, and adverse effects of ginger supplementation in patients with FD.

**Methods:**

This open-label clinical trial was conducted at the Internal Medicine Outpatient Department, University of Debrecen. This study was conducted in full compliance with the ethical principles outlined in the Declaration of Helsinki. The study protocol was reviewed and approved by the Ethics Committee of the University of Debrecen (registry reference number: DE RKEB/IKEB 5622-2020). All participants provided written informed consent prior to their inclusion in the study. Fifty patients with FD were initially enrolled, and 47 participants completed the study. Ginger supplementation was administered at a dose of 1080 mg/d in divided doses over 8 weeks. Adverse effects were assessed weekly through clinical evaluations and self-reports, and tolerability was rated by participants at the end of the trial.

**Results:**

The study included 47 patients with FD who completed the trial, with a mean (SD) age of 51.49 (14.64) years. Of the participants, 78.7% were females. Ginger supplementation was well tolerated, with mild and transient adverse effects reported, including bloating (14.9%), heartburn (12.8%), and diarrhea (10.6%). None of these adverse events necessitated discontinuation of the treatment. Tolerability was rated as good or excellent by 87.2% of participants, and no severe adverse events were observed.

**Conclusions:**

Preliminary findings suggest ginger is well tolerated and may be a viable complementary dietary therapy, though further research is needed. ClinicalTrials.gov identifier: NCT06313814.

## Graphical abstract

**Figure:**
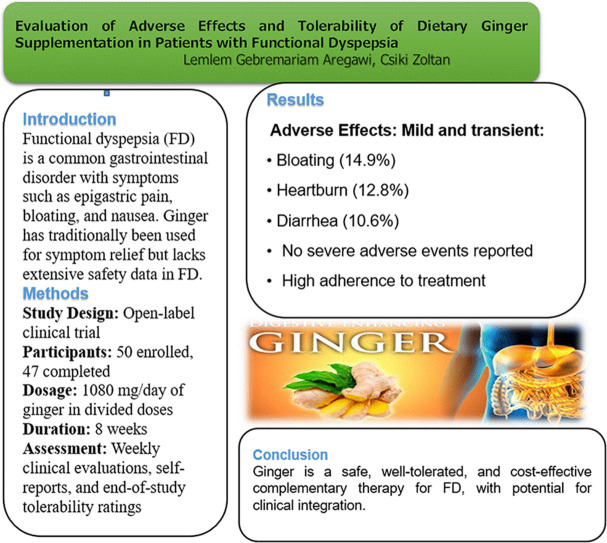

## Introduction

Functional dyspepsia (FD) is a common gastrointestinal disorder, affecting up to 20% of the population worldwide. It is characterized by persistent or recurrent upper abdominal discomfort or pain, early satiety, bloating, and nausea in the absence of structural or biochemical abnormalities. It is categorized into 3 subtypes: epigastric pain syndrome, postprandial distress syndrome, and a mixed form, each believed to arise from distinct mechanisms. Diagnosis relies on the Rome IV criteria, which describe FD as the presence of 1 or more symptoms—such as epigastric pain, burning, early satiety, or postprandial fullness in the absence of structural abnormalities detectable through imaging or endoscopy.^1^ Despite its prevalence, the pathophysiology of FD is not fully understood, though factors such as delayed gastric emptying, visceral hypersensitivity, and psychosocial influences are considered contributory. Functional dyspepsia not only impairs quality of life but also places a significant economic burden on healthcare systems.^2^

Current treatments for FD, including proton pump inhibitors, prokinetics, and antidepressants, often show inconsistent efficacy and are associated with undesirable side effects.^3^ As a result, there is growing interest in complementary therapies such as herbal medicines.^4^ Ginger supplementation, derived from the rhizome of Zingiber officinale, has gained recognition in food and nutrition due to its bioactive compounds, such as gingerols and shogaols, which exhibit antioxidant, anti-inflammatory, and digestive health properties. As a natural dietary supplement, ginger is widely used to alleviate gastrointestinal discomfort, including nausea, bloating, and indigestion, making it particularly valuable in managing FD and related disorders. Its incorporation into nutritional strategies aligns with the growing emphasis on functional foods that promote health and prevent disease. Ginger’s versatility allows it to be consumed in various forms—powder, capsules, extracts, or teas—making it an accessible and culturally adaptable remedy. Furthermore, its role in improving appetite, enhancing metabolic processes, and potentially modulating gut microbiota highlights its multifaceted benefits in modern nutrition science.^5^

Studies suggest that ginger may improve gastric motility and reduce bloating and nausea, which are hallmark symptoms of FD.^6^ Ginger is frequently suggested for gastrointestinal problems (such as flatulence, cramps, diarrhea, and spastic colon), and is also used to treat headaches, nausea, and motion sickness.^7^ Ginger root has many different nutrients, including para-aminobenzoic acid, manganese, choline, folic acid, inositol, para-aminobenzoic acid, various B vitamins, and vitamin C, in addition to essential oils.^8^ Because ginger encourages the release of bile from the gallbladder, it is contraindicated for patients with cholecystitis or cholangitis. Ginger also interacts with warfarin^9^ and potentially other anticoagulant drugs. Additionally, it might interfere with the class of drugs known as cyclophosphamides, which are used to treat autoimmune illnesses, multiple sclerosis, breast and testicular cancer, Hodgkin’s and non-Hodgkin’s lymphoma, and leukemia. By stimulating muscarinic receptors and blocking Ca^++^ channels, aqueous ginger extract appears to have a dual inhibitory effect that lowers blood pressure.^10^^,^^11^ Some researchers think ginger offers a portion of undiscovered medical benefits and side effects; thus, they recommend more research on this dietary herbal.^5^

Although several studies suggest that ginger may improve gastrointestinal symptoms, there is limited evidence regarding its safety and tolerability in patients with gastrointestinal disorders like FD. Concerns such as gastrointestinal discomfort, potential bleeding risks (due to its antiplatelet effects), and interactions with conventional FD medications have been sporadically reported.^2^ Addressing this gap is vital for ensuring the safe and effective integration of ginger supplements as a complementary therapy for FD. Therefore, this study aims to evaluate the adverse effects and tolerability of ginger supplementation in patients with FD. The findings may provide valuable insights into the safety and applicability of ginger as a complementary therapy for FD management.

## Materials and Methods

### Study design

This was an open-label clinical trial conducted at the Internal Medicine Outpatient Department of the University of Debrecen. Ethical approval was obtained from the institutional review board of the University of Debrecen (reference number: DE RKEB/IKEB 5622-2020), and informed consent was obtained from all participants before enrollment. The trial is registered at ClinicalTrials.gov (NCT06313814).

### Participants

Patients aged 18 to 72 years who met the Rome IV criteria for FD were included in the study. Inclusion criteria were (1) adult patients aged 18 to 72 years; (2) diagnosed with FD on the basis of Rome IV criteria; (3) symptomatic for at least 3 months before the study; and (4) not on any conflicting medications (such as proton pump inhibitors or H2 receptor antagonists) that may interfere with the trial outcomes. Exclusion criteria were (1) patients with organic gastrointestinal disorders (eg, peptic ulcer disease, gastroesophageal reflux disease, or malignancy); (2) pregnant or breastfeeding women; (3) history of allergic reactions to ginger or related supplements; and (4) patients taking medications known to interact with ginger (eg, anticoagulants).

### Randomization and masking

In a single-center, open-label clinical trial, given that our study was a single-group clinical study, to minimize bias and ensure validity, where there’s only 1 treatment group (in our case, patients received ginger supplementation), the conventional randomization to allocate participants to different treatment arms and masking to conceal the treatment assignment from participants and researchers might not be applicable in the traditional sense. However, we applied some measures to minimize bias and enhance the rigor in this study. (1) Randomization of treatment timing: Although all participants received ginger supplementation, we randomized the timing of when they started receiving the supplementation. This could help control for any temporal effects or other confounding factors. (2) Standardized procedures: The procedures for data collection, assessment, and analysis were standardized across all participants and could help minimize bias and enhance our study’s reliability. Although these measures cannot fully replicate the rigor of the trial with masking, they can still strengthen the internal validity of our study and provide valuable insights into the effects of ginger supplementation on quality of life in patients with FD.

### Intervention

All enrolled patients received ginger capsules (1080 mg/d) in divided doses for 8 weeks. The ginger supplement was standardized to contain 5% gingerols and was administered with meals to reduce the risk of gastrointestinal discomfort. No blinding was implemented in this trial, as patients were fully aware of the supplementation being provided. Supplementing with ginger is provided following the established protocol. For 8 weeks, the patients who were enrolled were instructed to take 2 capsules, each containing 540 mg. Participants were advised to carry on with their regular activities and daily eating routines throughout the investigation.

#### Swanson Ginger Root 540 mg supplement

Swanson Ginger Root 540 mg sounds like a comprehensive supplement for digestive health and overall wellness. Below is a breakdown of its main features.

##### Main ingredient

Ginger root (*Zingiber officinale*) powder, standardized to contain a minimum of 4% volatile oils. Ginger has been traditionally used for its various health benefits, particularly for digestive support and anti-inflammatory properties.

##### Dosage

Each capsule contains 540 mg of ginger root powder, equivalent to 1.35 g of fresh ginger root. The supplement dosage was 2 capsules per day taken with water, preferably with a meal. This dosage provides flexibility for users to adjust according to their needs.

##### Capsule material

The capsules are made from gelatin derived from bovine sources. Gelatin capsules are commonly used in dietary supplements and are generally well tolerated by most individuals. However, it is important to note for those who follow specific dietary restrictions or have religious beliefs that prohibit the consumption of bovine-derived products.

##### Free from common allergens

The product is free from artificial colors, flavors, and preservatives, making it suitable for those who prefer natural supplements. Additionally, it is free from common allergens such as wheat, peanuts, eggs, fish, tree nuts, soy, milk, and shellfish, which is beneficial for individuals with food sensitivities or allergies.

### Outcome measures

Primary outcomes included the incidence of adverse effects, assessed through weekly clinical follow-up visits and patient self-reports. Secondary outcomes included patient-rated tolerability, scored using a 5-point Likert scale (excellent, good, moderate, poor, and very poor).

### Statistical analysis

Statistical analysis was performed by SPSS version 26.0 (IBM, Armonk, New York). Descriptive statistics were used to summarize demographic characteristics, the frequency of adverse effects, and tolerability ratings. Paired *t*-tests were used to compare baseline and postintervention dyspeptic symptom scores. A significance level of *P* < 0.05 was considered statistically significant.

## Results

### Participant characteristics

Fifty patients were enrolled, and 47 completed the study (94% completion rate) (Figure). The mean (SD) age of participants was 51.49 (14.64) years, and 78.8% were female (Table 1).

**Figure:**
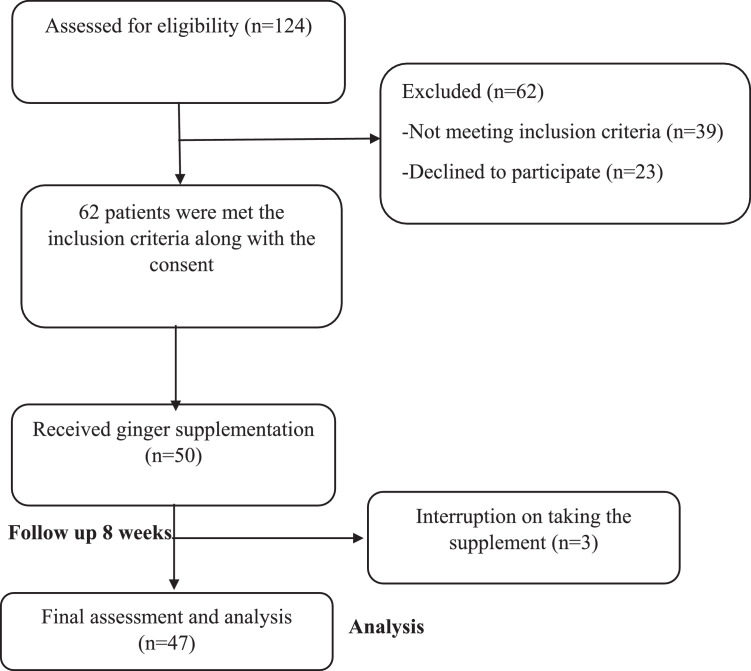
Flow diagram of study subjects.

**Table 1:** Baseline characteristics of enrolled subjects (n = 47).

Parameters	Values
Gender*	
Male	10 (21.3)
Female	37 (78.7)
Age (y)†	51.49 (14.64)
BMI (kg/m^2^)	25.83 (5.03)

BMI = body mass index.

⁎ Gender is presented as number (n) and percentage (%).

† Age and BMI are presented as mean (SD).

### Adverse effects

The most commonly reported adverse effects were mild bloating (14.9%), heartburn (12.8%), and diarrhea (10.6%). These adverse effects were transient and self-limiting. None of the patients discontinued ginger supplementation due to adverse effects, and no serious adverse events were reported. One patient reported a mild headache, which resolved without intervention (Table 2).

**Table 2:** Adverse effect.

Parameters	Frequency (n = 47)	Percentage
	n	%
Bloating	7	14.9
Heartburn	6	12.8
Diarrhea	5	10.6
Headache	1	2.1

### Tolerability

At the end of the study, 87.2% of patients rated the tolerability of ginger supplementation as good or excellent. No patient-rated tolerability as poor or very poor (Table 3).

**Table 3:** Tolerability rating.

Parameters	Frequency (n = 47)	Percentage
	n	%
Excellent	18	38.3
Good	23	48.9
Moderate	6	12.8
Poor	0	0
Very poor	0	0

## Discussion

Functional dyspepsia, characterized by chronic upper abdominal discomfort, affects quality of life significantly. Ginger (*Zingiber officinale*) is a natural dietary remedy used for its anti-inflammatory and prokinetic effects. This study evaluates the safety and tolerability of ginger supplementation in patients with FD based on findings where mild bloating (14.9%), heartburn (12.8%), and diarrhea (10.6%) were the most reported adverse effects. Additionally, 87.2% of patients rated tolerability as good or excellent, with no reports of poor tolerability.

The occurrence of mild bloating in 14.9% of participants reflects similar findings in other clinical trials. In a randomized controlled trial, 12% of participants reported bloating during ginger supplementation.^5^ This might be attributed to ginger’s prokinetic effect, which may transiently disrupt gastric motility in sensitive individuals^12^ noted a similar prevalence of bloating, emphasizing its mild and self-limiting nature.

Heartburn occurred in 12.8% of participants in this study, consistent with findings from previous research. This side effect is likely attributable to ginger’s capacity to stimulate gastric acid secretion, driven by its bioactive compounds gingerols and shogaols, which can irritate the gastric mucosa and exacerbate reflux symptoms. Lete and Allué^13^ reported that 10% to 15% of participants experienced heartburn at doses exceeding 1 g/d, reinforcing the dose-dependent nature of this adverse effect. Similarly, a systematic review identified heartburn as a common, dose-related side effect of ginger supplementation.^14^ Ginger has been found to relax the lower esophageal sphincter, which is the primary mechanism behind reflux^15^; however, minor heartburn may be improved with ginger consumption, and may actually be attributable to a positive shift in the composition and function of gastrointestinal microbiota.^16^

The 10.6% incidence of diarrhea in this study mirrors findings in previous trials but remains lower than the adverse effects reported for synthetic prokinetics. A similar prevalence of diarrhea in participants consuming high-dose ginger (2 g/d).^17^ A study compared herbal remedies and noted ginger’s mild laxative effect as a common, though manageable, side effect.^18^ Incidence may depend on individual gastrointestinal sensitivity and the preparation method of ginger.

### High tolerability ratings (87.2%)

The majority (87.2%) of participants rated ginger supplementation as good or excellent, aligning with findings from previous studies.^13^ A study showed that over 85% of participants reported positive tolerability, even with mild adverse effects^14^ Lete and Allué^13^ found similarly high satisfaction ratings in their study on ginger for gastrointestinal disorders. This suggests that benefits outweigh mild adverse effects for most patients. No participants rated tolerability as poor or very poor, emphasizing ginger’s overall acceptance. Similar findings in clinical trials reinforce its status as a well-tolerated natural remedy and reported no cases of poor tolerability in their trial, highlighting the minimal severity of side effects.^5^ In comparative studies^12^ ginger consistently showed better tolerability than conventional pharmacological agents.

Gender imbalance was observed in this study, with a higher proportion of female participants (78.7%). This demographic distribution reflects the higher prevalence of FD among women, which has been consistently reported in epidemiological studies.^13^ However, we recognize that this may limit the generalizability of our findings to male patients with FD.

Sex-related differences in gastrointestinal physiology, symptom perception, and metabolism could potentially influence the tolerability and response to ginger supplementation. For instance, studies suggest that variations in gastric emptying rates, hormonal influences, and pain sensitivity may differ between males and females, potentially affecting both the experience of FD symptoms and the response to dietary interventions.^19^^,^^20^ Additionally, differences in enzyme activity related to ginger metabolism could play a role, though specific data on sex-based pharmacokinetics of ginger compounds remain limited.^13^^,^^19^ Given these differences, the study's findings may not be directly applicable to male patients with FD. Future research should include a more balanced gender distribution to evaluate potential sex-specific responses to ginger supplementation.

## Strengths and Limitations of the Study

### Strengths

#### Focus on tolerability and safety

Unlike most previous studies that primarily assess the efficacy of ginger, this study specifically evaluates its tolerability and potential adverse effects in patients with FD, providing valuable insights for clinical practice.

#### Standardized assessment methods

The study employed structured questionnaires and standardized procedures to assess tolerability and adverse effects, ensuring consistency in data collection.

#### Monitoring of adverse events

Participants were closely monitored throughout the study period, with physician evaluations complementing self-reported outcomes to minimize bias.

#### Real-world applicability

By using an open-label design, the study reflects a real-world setting where patients are aware of their treatment, which can enhance the external validity of the findings.

#### Contribution to existing literature

The study adds to the growing body of research on ginger as a complementary therapy for FD, particularly in terms of safety and acceptability, which are crucial for long-term adherence.

### Limitations

#### Lack of a control group

The absence of a placebo or alternative treatment group limits the ability to determine whether the observed tolerability and mild adverse effects are specific to ginger supplementation or part of natural variations in FD symptoms.

#### Open-label design and potential bias

Since participants were aware that they were receiving ginger supplementation, their perceptions of tolerability and adverse effects may have been influenced by expectations, introducing potential bias.

#### Gender imbalance

The majority of participants were female (78.7%), which may limit the generalizability of findings to male patients, as sex-related differences in gastrointestinal response and ginger metabolism could play a role.

#### Small sample size

A small sample size may limit the generalizability of the findings. Future studies should increase the sample size to ensure more reliable results and improve the generalizability of the findings.

#### Reliance on self-reported data

Although physician evaluations were included, tolerability ratings were primarily self-reported, which introduces subjectivity and potential recall bias.

## Implications for Clinical Practice

The findings highlight the potential of dietary ginger as a safe and effective option for managing FD, with significant implications for clinical practice. Clinically, they support the use of ginger as a natural, low-cost alternative or adjunct to conventional therapies for FD, particularly for patients seeking complementary treatment options. The high tolerability and safety of ginger may improve patient adherence and satisfaction with treatment regimens. From a research perspective, these results pave the way for further investigations to confirm the benefits in larger and more diverse populations, optimize dosage, and evaluate long-term safety and efficacy. Public health efforts could leverage ginger’s accessibility to improve FD management, especially in resource-limited settings where access to conventional medications may be constrained.

## Declaration of competing interest

None.
